# Machine Learning-Based Risk Prediction of Discharge Status for Sepsis

**DOI:** 10.3390/e26080625

**Published:** 2024-07-25

**Authors:** Kaida Cai, Yuqing Lou, Zhengyan Wang, Xiaofang Yang, Xin Zhao

**Affiliations:** 1 School of Public Health, Southeast University, Nanjing 210009, China; 2 School of Mathematics, Southeast University, Nanjing 210009, China; 213211263@seu.edu.cn (Y.L.); zhengyanwang@seu.edu.cn (Z.W.); xiaofangyang@seu.edu.cn (X.Y.); 3 Key Laboratory of Measurement and Control of Complex Systems of Engineering, Ministry of Education, Southeast University, Nanjing 210096, China

**Keywords:** machine learning, feature selection, information gain, missing data imputation, sepsis

## Abstract

As a severe inflammatory response syndrome, sepsis presents complex challenges in predicting patient outcomes due to its unclear pathogenesis and the unstable discharge status of affected individuals. In this study, we develop a machine learning-based method for predicting the discharge status of sepsis patients, aiming to improve treatment decisions. To enhance the robustness of our analysis against outliers, we incorporate robust statistical methods, specifically the minimum covariance determinant technique. We utilize the random forest imputation method to effectively manage and impute missing data. For feature selection, we employ Lasso penalized logistic regression, which efficiently identifies significant predictors and reduces model complexity, setting the stage for the application of more complex predictive methods. Our predictive analysis incorporates multiple machine learning methods, including random forest, support vector machine, and XGBoost. We compare the prediction performance of these methods with Lasso penalized logistic regression to identify the most effective approach. Each method’s performance is rigorously evaluated through ten iterations of 10-fold cross-validation to ensure robust and reliable results. Our comparative analysis reveals that XGBoost surpasses the other models, demonstrating its exceptional capability to navigate the complexities of sepsis data effectively.

## 1. Introduction

Sepsis, characterized as a severe inflammatory response syndrome, is a critical medical condition that arises from an unbalanced reaction of the body to infection. Triggered primarily by bacterial infections, sepsis can also result from viral or fungal pathogens [1]. The clinical presentation of sepsis is highly variable, encompassing symptoms such as fever, chills, increased heart rate, rapid breathing, low blood pressure, and alterations in consciousness, which can escalate into severe sepsis and result in multiple organ failure impacting the respiratory, cardiovascular, renal, hepatic, and central nervous systems [2]. Despite the prevalence and severity of sepsis, the exact mechanisms underlying its onset and progression remain poorly understood, posing significant challenges in its management and treatment [3]. The complexity of sepsis, coupled with its diverse manifestations, makes it difficult to predict and manage [4]. Current strategies often focus on the rapid identification and treatment of the underlying infection and support of organ function [5]. However, the need for early and accurate prediction of patient outcomes, such as discharge status, has become increasingly apparent. This predictive capability is crucial for evaluating the therapeutic efficacy of different treatment approaches and for assessing the long-term prognosis of patients [6].

Risk prediction of discharge status, which categorizes outcomes into survival or death, utilizes current discharge status information along with various monitored clinical signals. By predicting discharge status, healthcare professionals can better anticipate the subsequent development of a patient’s condition and the effectiveness of the treatment provided, thereby facilitating more targeted diagnosis, treatment, and planning of medical resources [7]. However, the prediction task is complicated by the high dimensionality of clinical features, significant variability among patients, and the presence of missing data in medical datasets. Machine learning methods have proven effective in navigating these complexities, offering new tools for early detection and accurate prediction of sepsis outcomes [8,9]. Studies have shown that machine learning models significantly outperform traditional methods such as the SAPS II score in predicting mortality among sepsis patients [10]. In the context of machine learning, feature selection becomes paramount as it directly influences the model’s performance by eliminating redundant or irrelevant data, thus enhancing the model’s predictive accuracy and reducing computational costs [11]. Effective feature selection not only improves model interpretability but also mitigates overfitting, particularly in scenarios with high-dimensional data spaces common in clinical settings [12]. In such environments, regularization methods like least absolute shrinkage and selection operator (Lasso) are particularly valuable [13]. These techniques enhance model generalizability by selecting meaningful features through penalization of coefficients’ complexity. Lasso promotes sparse solutions by imposing an L1 penalty, effectively reducing the number of features and the model’s complexity [13]. Moreover, dealing with missing data is a critical step in the predictive modeling process, especially in healthcare where datasets often contain gaps due to various reasons ranging from equipment malfunction to inconsistent data entry practices [14,15]. Advanced imputation techniques, such as sophisticated algorithms like random forest imputation, are employed to estimate missing values, ensuring the integrity and usability of the dataset for building robust predictive models [16]. These strategies of feature selection and missing data imputation play a crucial role in the preparation of the dataset, setting the foundation for the deployment of machine learning algorithms that are capable of handling the intricacies and variability inherent in medical data [17].

Recent research into sepsis has increasingly emphasized the utilization of advanced methodologies to improve predictive accuracy and patient outcomes. For instance, support vector machines are applied to predict severe cases of sepsis, providing a detailed analysis of the algorithm’s effectiveness in forecasting the severity of the condition [18]. In the context of sepsis management, sophisticated machine learning approaches have been shown to significantly improve the accuracy of short-term outcome predictions, such as 30-day mortality rates, highlighting their potential over conventional models [19]. In our discussion on predictive modeling for sepsis, it is crucial to acknowledge the role of survival analysis, a method that has been refined and extensively applied in medical research [20]. Particularly in sepsis research, survival analysis techniques have been instrumental in evaluating how clinical practices influence patient outcomes [21]. The focus of our research is to compare traditional statistical methods and novel machine learning techniques to establish the most effective approach for predicting the discharge status of sepsis patients. By integrating advanced machine learning-based imputation methods to manage missing data and applying sophisticated feature selection techniques, we aim to enhance the predictive accuracy of our models and ensure their adaptability to the individual variations in patient profiles. In conclusion, our work seeks to advance sepsis management by developing a reliable machine learning-based predictive model for patient discharge status. This model aims to support clinical decision-making processes, enhance the treatment and management of sepsis, and ultimately improve patient care outcomes in diverse healthcare settings.

The rest of this work is organized as follows. The data preprocessing, including outlier detection and missing data imputation, and baseline data analysis are conducted in Section 2. Section 3 introduces the feature selection. The comparison of different machine learning-based risk prediction methods and the corresponding analysis results are given in Section 4. Section 5 includes discussions and conclusions. All computations are implemented using the R programming language.

## 2. Data Preprocessing and Baseline Data Analysis

The real data are cross-section data from the medical information mart for intensive care (MIMIC)-III database, which includes retrospectively collected critical care data of over 40,000 patients admitted to intensive care units at the Beth Israel Deaconess Medical Center (BIDMC) [22,23]. It contains clinical information from 6273 sepsis patients. After removing features with no valid informational content, such as patient IDs and ICU IDs which do not contribute to the analysis, there exist 6 categorical features and 115 numerical features in our dataset. Among these features, 12 of them are baseline data collected at the beginning of treatment. They provide baseline values for the health status, physiological parameters, and other basic information of patients before treatment. The rest of the features are clinical data collected from the patients’ first day of admission. Specifically, the dataset includes clinical measurements such as hemoglobin, hematocrit, and platelet counts, which are critical for diagnosing and monitoring the progression of sepsis. We also analyze biochemical markers like albumin, globulin, and electrolytes to assess the metabolic status of patients. Furthermore, vital signs such as heart rate, blood pressure, and respiratory rate are considered, which are essential for the clinical management of sepsis patients. In this work, we define the discharge status of the patients, a binary variable, as the response feature. All other features are considered as covariates for constructing the prediction method. To enhance the clarity and accessibility of our data presentation, each feature used in this work has been assigned a unique feature notation. The corresponding descriptions for these notations are detailed in the accompanying Table 1. To briefly show the information of the numeric baseline data, a five-number summary of the numeric features in the baseline data is shown in Table 2. In our dataset, both features X5 (length of stay in hospital) and X6 (length of stay in ICU) are measured in days. The presence of a negative value for feature X5 and an abnormally low weight of 1 for feature X1 in our dataset are identified as data entry errors. In this study, such anomalies are treated as outliers and handled appropriately in our data processing steps. Some features are categorical, such as features X9 and X16. Specifically, X9 includes 8 distinct categories, and X16 comprises 27 distinct categories. To handle these categorical features, we employ factorization to ensure they are appropriately processed during our analyses. This approach enables us to maintain the integrity of categorical data and facilitates more accurate statistical modeling.

### 2.1. Outlier Detection and Missing Data Imputation

Outliers are defined as data points that significantly deviate from other observations, displaying values that are extraordinarily high or low. Detecting outliers in baseline data is more straightforward due to its predictable nature, unlike the data from the initial day, which does not have a standardized method for identifying outliers. Therefore, we only evaluated numerical features in the baseline data for outliers. In our study, we utilized the isolation forest method, which is effective for detecting anomalies in multi-dimensional data [24]. We use 10-fold cross-validation to determine the optimal probability threshold for outlier detection, which is set at 0.391. With this threshold, we train the isolation forests and identify a total of 1473 outliers in our multivariate dataset. In this study, a robust data processing method is applied to mitigate outlier effects. The robust preprocessing method primarily utilizes the minimum covariance determinant (MCD) for robust estimation [25]. The MCD is a highly effective robust statistical technique used to identify and mitigate the impact of outliers in data [25,26]. Additionally, all numeric features are standardized to have zero mean and unit variance.

There is a large amount of missing data in the dataset. Table 3 summarizes the missing data rates across clinical features and patient records. Within each missing rate interval, the Frequency column shows the number of features or patient measurement records with missing data, while the Percentage column indicates the proportion of total features or records. As shown in Table 3, 34 clinical features have missing rates of 40% or higher, and more than 591 patient measurement records also exhibit missing rates at or above 40%. Retaining observation features with a large amount of missing data is not conducive to statistical analysis and inference [27]. Moreover, applying statistical methods to impute a large number of missing data may easily lead to serious bias in statistical analysis results, thereby reducing the quality and reliability of the data [28]. In this work, we remove the features with missing rates exceeding 40% and patient data with missing data counts greater than or equal to 40.

**Figure 1:**
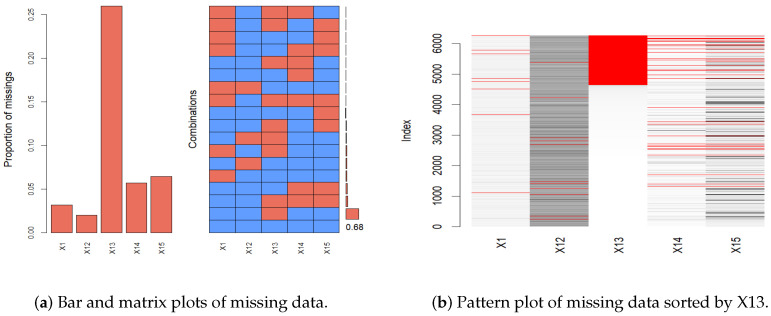
The plots of the missing patterns.

A bar plot, matrix plot, and pattern plot of missing data are shown in Figure 1, which visually represents the distribution of missing data across various features in our dataset [15,29]. Given the extensive number of features in our dataset, we carefully selected five representative features to illustrate the missing data patterns, thus avoiding overly complex visualizations. The selected features provide a clear view of the missingness across the dataset without compromising the understanding of its structure. As shown in Figure 1, the bar plot and matrix plot indicate the proportion and interrelationships of missing data across features. From the bar and matrix plots of missing data, it is observable that the proportion of missingness across each feature exhibits no discernible pattern, suggesting that the missing data are missing completely at random (MCAR). The right side of Figure 1 showcases a missing data pattern plot sorted by feature X13 to examine potential correlations and patterns among features. In this plot, there exist overlapping red areas between features X14 and X15, suggesting that instances of missing data in these features frequently coincide. The co-occurrence of missing values in these features suggests that their missingness is influenced by the observed values in other features. This pattern aligns with a missing at random (MAR) assumption. Overall, the observed patterns indicate a mix of MCAR and MAR mechanisms. According to Stekhoven and Bühlmann [16], random forest imputation is proven to be effective for MCAR and MAR data, providing high accuracy and maintaining data integrity without introducing bias. It does not require any assumptions and specific data types [16,30]. In this work, we employ the missRanger R package (R version 4.3.2), an efficient implementation of random forest imputation. This method iteratively builds random forest models to impute missing data by learning from observed patterns in the dataset [31]. By leveraging missRanger, we capitalize on its advantages of enhanced speed and robustness, significantly improving the handling of missing data in our analyses.

### 2.2. Baseline Data Analysis

**Figure 2:**
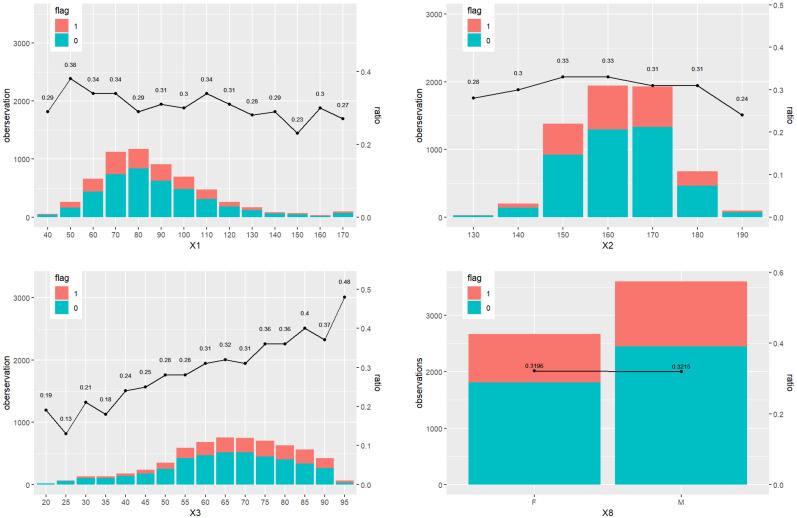
The baseline information for status 0 (survival) and 1 (death), including weight (X1), height (X2), age (X3), and gender (X8). The ratio is the percentage of status 1 over all the observations across all the patients.

We study the association between patient discharge status and the main baseline features, including weight (X1), height (X2), age (X3), and gender (X8). To provide a preliminary exploration of potential patterns in our data, we present the results of the descriptive statistical analysis in Figure 2. The subfigure for weight shows that the proportion of in-hospital mortality approximately hovers around 0.3, without significant variation. Similarly, the height analysis reveals a generally stable mortality rate across most heights, with some deviations at extreme values. The age analysis indicates a clear trend where in-hospital mortality increases with age, reflecting a higher risk among older patients. Lastly, the gender comparison reveals minimal differences in mortality rates, suggesting a negligible impact of gender on discharge outcomes.

Then we conduct the differential analysis for all baseline features and patient discharge status. The categorical features in the baseline data include gender (X8), ethnicity (X9), first hospitalization (X10), first ICU admission (X11), and patient disease (X16). Among them, some categories of X16 appear fewer than five times. Fisher’s exact test is particularly suited for cases like feature X16, where small sample sizes result in expected frequencies being less than five. It is recommended over the chi-squared test in such scenarios because it does not depend on the large-sample conditions that the chi-squared test requires [32]. Therefore, due to its robustness in handling small sample sizes, Fisher’s exact test is employed for X16. We obtain the *p*-value of Fisher’s exact test is about 0.0005, indicating a significant association between X16 and discharge status. This suggests that the observed data of X16 are unlikely to have occurred randomly but rather due to a true association. Then, the chi-square test is conducted on the rest of the categorical features and patient discharge status. The results of the chi-square test in Table 4 suggest that the *p*-values of X8 and X10 are greater than 0.05, indicating that they are independent of patient discharge status. On the contrary, *p*-values of X9 and first X11 are less than 0.05, indicating that these two categorical features are not independent of patient discharge status.

For each numerical feature in the baseline data, we divide the data into two groups based on the discharge statuses of survival and death. We then assess the normality of these groups using the Shapiro–Wilk test to ensure that the assumptions underlying the *t*-test are met. The results, detailed in Table 5, reveal that the data do not conform to normal distribution, indicating that the *t*-test may not be appropriate for this analysis. Consequently, we consider the Wilcoxon rank-sum test, a non-parametric method which does not require the normality assumption for valid results [33]. The test results in Table 5 show that *p*-values are all less than $2.2\times 10^{-16}$, suggesting that there exist significant differences in the numerical features of baseline data between the two discharge statuses of patients.

The above exploratory baseline data analysis show that there is an association between the baseline data and patient discharge status. Also, the numerical features do not follow the normal distribution assumption. However, exploratory data analysis cannot provide more specific information about the association between features of baseline data and patient discharge status, so further analysis is needed.

## 3. Feature Selection

To effectively detect multicollinearity, we calculate the generalized variance inflation factor (GVIF) and the adjusted generalized variance inflation factor (AGVIF) for all features. The results are summarized in Table 6. This table categorizes the features into different VIF ranges, indicating the number of features and their percentage of the total within each range. From the table, it is evident that a significant proportion of features, particularly those with GVIF and AGVIF values exceeding 5, demonstrate severe multicollinearity issues. Specifically, features in the GVIF range of (5,+∞] account for 55.814% and those in the same range for AGVIF make up 9.032%. This high prevalence of elevated VIF values suggests substantial multicollinearity, which may compromise the stability and accuracy of the model.

To address multicollinearity issues and select significant features, we employ the Lasso penalized method and the information gain feature selection method based on a logistic regression model [13,34,35]. To compare the performance of the two methods, we utilize accuracy (ACC), ROC curves, and AUC values, all of which are calculated based on 10-fold cross-validation. This approach ensures a rigorous model evaluation and helps to prevent overfitting by systematically validating the methods across different subsets of data. We define death and survival as the positive and negative discharge status, respectively. Thus, the number of true positive samples (TP) indicates the number of samples correctly identified as death among those actually dead. The number of false positive samples (FP) indicates the number of samples incorrectly identified as death among those who actually survived. The number of true negative samples (TN) indicates the number of samples correctly identified as survival among those who actually survived. The number of false negative samples (FN) indicates the number of samples incorrectly identified as survival among those actually dead. Then the formula of ACC is defined as follows

(1) $$\mathrm{ACC}=\frac{\left(\mathrm{TP}+\mathrm{TN}\right)}{\left(\mathrm{TP}+\mathrm{FP}+\mathrm{TN}+\mathrm{FN}\right)}.$$

**Figure 3:**
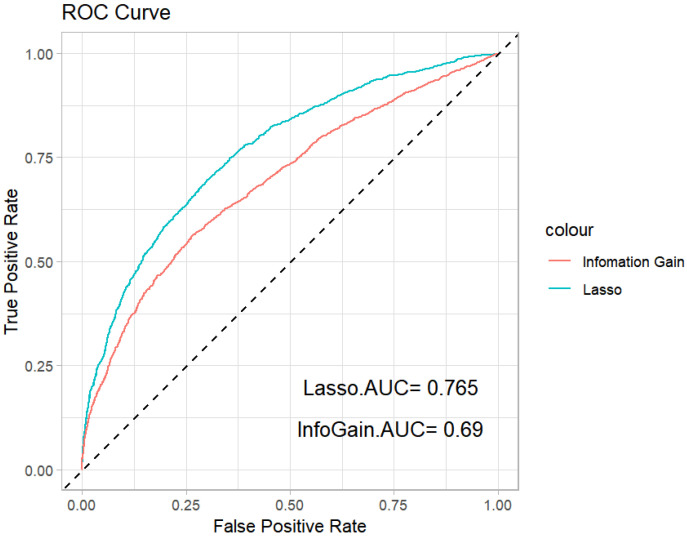
ROC curves of the Lasso penalized method and information gain feature selection method.

The ROC curves and corresponding AUC values are shown in Figure 3. The values of the average accuracy and AUC are shown in Table 7. We can see that the Lasso method has the largest average accuracy and AUC values. Therefore, we conclude that the Lasso penalized logistic regression performs better than the information gain method.

## 4. Machine Learning-Based Risk Prediction

Three different machine learning methods are employed, including random forest, support vector machine, and XGBoost, to establish risk prediction model of discharge status for sepsis. Random forest, an ensemble learning model consisting of multiple decision trees, leverages a majority voting mechanism to improve the generalization of individual trees for binary classification [36]. SVM is employed to find an optimal hyperplane that best separates the two classes in our binary classification problem [37]. XGBoost, an advanced form of gradient boosting, uses a second-order Taylor expansion of the loss function and includes a regularization term to enhance model accuracy and prevent overfitting [38]. To establish an effective risk prediction model, we compare the prediction performance of these methods with Lasso penalized logistic regression (Logit). Each method is evaluated based on its predictive performance, utilizing the discharge status and significant features as the response and predictor features, respectively. This comparative analysis helps identify the most efficient risk prediction approach of discharge status for sepsis.

**Figure 4:**
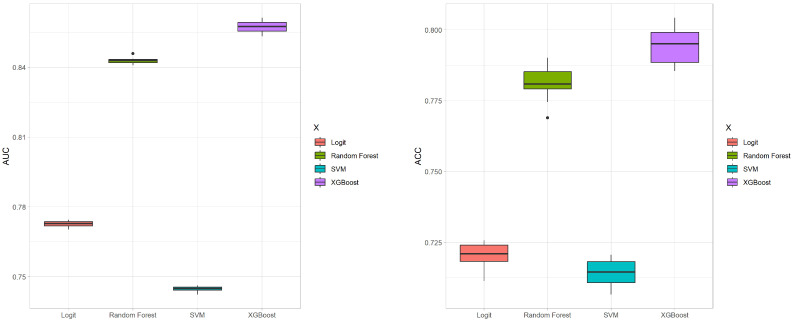
Box plots of AUC and ACC values for the four methods with 10-fold cross-validation.

**Figure 5:**
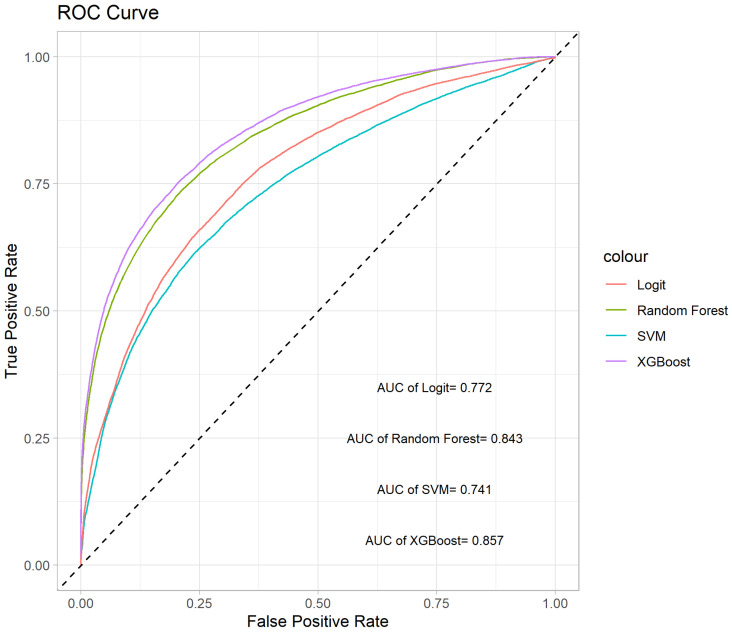
ROC curves of the four methods.

We conduct 10 iterations of 10-fold cross-validation to evaluate the four methods, focusing on their average AUC and ACC values. These results are illustrated in the box plots shown in Figure 4. The box plot for the AUC values indicates that XGBoost and random forest significantly outperform SVM and Logit in terms of predictive accuracy. Moreover, the variability among the four methods is relatively small. In terms of ACC values, XGBoost provides the highest mean accuracy. The ROC curves for the four methods are depicted in Figure 5, showing that the curves of XGBoost and random forest generally surpass those of the other methods. Also, the curve of XGBoost is slightly higher than that of random forest, which indicates its overall highest AUC value.

The average results of confusion matrices for each method are displayed in Table 8. XGBoost exhibits the best predictive performance with the lowest misclassification rate for cases where actual survival is incorrectly predicted as death. When actual death is misclassified as survival, the misclassification rates are comparable across all methods, with XGBoost registering the lowest rate. Overall, the XGBoost method demonstrates superior performance across various metrics on this sepsis dataset.

## 5. Discussion and Conclusions

Based on machine learning methods, a prediction mechanism of the discharge status for sepsis is proposed in this work. We apply the proposed mechanism to analyze a real cross-section data of sepsis from the MIMIC-III database. A machine learning method, the random forest imputation method, is used to conduct the missing data imputation. In this work, we analyze the baseline information data of sepsis patients by using differential analysis, chi-square test, and Wilcoxon rank-sum test. To deal with the multicollinearity issue, we employ the Lasso penalized method and information gain feature selection method based on logistic regression to select significant features. The feature selection can ensure that only significant features are included in the construction of the sepsis patient discharge status risk prediction model, thereby improving its reliability and effectiveness. Furthermore, three machine learning methods, including random forest, support vector machine, and XGBoost, are used to analyze the data with discharge status and significant features. We compare their predictive performance with that of Lasso penalized logistic regression. The superior performance of the XGBoost model demonstrates its potential as a valuable tool for predicting sepsis patient discharge status. In conclusion, the results of this study provide a stable and efficient machine learning-based model to predict sepsis patient discharge status. Regarding the trade-offs between model interpretability and predictive performance, we acknowledge the strengths of each approach in our discussion. XGBoost is recommended for scenarios prioritizing predictive accuracy due to its superior performance. Conversely, Lasso is more suitable for studies that require a deep understanding of the influence of each feature, as it provides clearer interpretability of model parameters.

In recognizing the limitations inherent in our study, it is crucial to note that our analysis is based on data from a single dataset. This constraint may limit the generalizability of our findings to other sepsis patient populations or clinical environments. To mitigate this limitation, we advocate for future studies to validate and replicate our results across a more diverse array of datasets, which could include data from different geographic regions and healthcare systems. In future studies, we also aim to methodically introduce interaction terms based on their statistical significance and clinical relevance. We will utilize advanced regularization methods, such as Elastic Net, to handle multicollinearity and explore the development of hierarchical models that systematically incorporate interaction terms. Additionally, considering the incorporation of survival analysis in future studies could further enhance our understanding of the long-term outcomes for sepsis patients.

## Figures and Tables

**Table 1:** The notations and interpretations of features.

Features	Interpretations
Y	Indicates patient status in the hospital: 0 for survival, 1 for death.
X1	Patient’s weight at ICU admission.
X2	Patient’s height.
X3	Patient’s age at ICU admission.
X4	Hospital stay sequence.
X5	Length of stay in hospital.
X6	Length of stay in ICU.
X7	Number of admissions the patient had to the ICU.
X8	Patient’s sex: F for female, M for male.
X9	Ethnicity of patients.
X10	Indicates if this was the patient’s first hospital admission: t for true, f for false.
X11	Indicates if this was the patient’s first ICU admission: t for true, f for false.
X12	Maximum of patient’s body temperature.
X13	Maximum of absolute monocyte.
X14	Maximum of prothrombin time.
X15	Maximum recorded value of the time indicator for the endogenous coagulation system.
X16	Patient disease; all patients in this dataset had sepsis.

**Table 2:** The five-number summary of the numeric features in the baseline data, including the minimum, lower quartile (QL), median, mean, upper quartile (QU), and maximum values.

No.	Min	QL	Median	Mean	QU	Max
X1	1.000	65.600	78.400	83.020	95.500	769.700
X2	122.000	160.000	170.000	168.800	178.000	203.000
X3	18.290	56.470	67.490	66.360	78.430	97.780
X4	1.000	1.000	1.000	1.679	2.000	28.000
X5	−0.704	7.025	13.394	19.937	24.371	1191.417
X6	0.010	2.250	4.900	8.098	10.170	375.940
X7	1.000	1.000	1.000	1.150	1.000	6.000

**Table 3:** Summary of missing rates.

Missing Rate Interval (%)	Feature			Record		
	**Frequency**	**Percentage (%)**		**Frequency**	**Percentage (%)**	
<40	87	71.901		5682	90.579	
≥40	34	28.099		591	9.421	

**Table 4:** Chi-square test of categorical features, except patient disease, and discharge status in baseline data.

Features	X-Squared	d	*p*-Value
X8	0.011	1	$9.149\times 10^{-1}$
X9	75.315	7	$1.238\times 10^{-13}$
X10	0.505	1	$4.771\times 10^{-1}$
X11	16.272	1	$4.021\times 10^{-5}$

**Table 5:** Results of normality test and rank-sum test of numerical features in baseline data.

Features	Shapiro-Wilk		Wilcoxon
	**0**	**1**	
X1	<$2.2\times 10^{-16}$	<$2.2\times 10^{-16}$	<$2.2\times 10^{-16}$
X2	<$2.2\times 10^{-16}$	$2.964\times 10^{-14}$	<$2.2\times 10^{-16}$
X3	<$2.2\times 10^{-16}$	$3.336\times 10^{-13}$	<$2.2\times 10^{-16}$
X4	<$2.2\times 10^{-16}$	<$2.2\times 10^{-16}$	<$2.2\times 10^{-16}$
X5	<$2.2\times 10^{-16}$	<$2.2\times 10^{-16}$	<$2.2\times 10^{-16}$
X6	<$2.2\times 10^{-16}$	<$2.2\times 10^{-16}$	<$2.2\times 10^{-16}$
X7	<$2.2\times 10^{-16}$	<$2.2\times 10^{-16}$	<$2.2\times 10^{-16}$

**Table 6:** Summary of VIF values for all features. GVIF and AGVIF represent the generalized variance inflation factor and adjusted generalized variance inflation factor, respectively.

GVIF	Frequency	Percentage (%)	AGVIF	Frequency	Percentage (%)
[0,1.6]	6	6.977	[0,1.6]	23	26.744
(1.6,2.2]	14	16.279	(1.6,2.2]	12	13.953
(2.2,3.2]	6	6.977	(2.2,3.2]	19	22.093
(3.2,5]	12	13.953	(3.2,5]	24	27.907
(5,+∞]	48	55.814	(5,+∞]	8	9.302

**Table 7:** The values of the average ACC and AUC for the Lasso penalized method and information gain feature selection method.

Method	ACC	AUC
Lasso	0.679	0.765
Information Gain	0.628	0.691

**Table 8:** The average results of the confusion matrix for four methods.

True Status	Logit			Random Forest			SVM			XGBoost	
	0	1		0	1		0	1		0	1
0	2782.3	1047.7		3064.4	765.6		2822.0	1008.0		3116.3	713.7
1	539.4	1312.6		477.2	1374.8		590.6	1237.1		453.3	1398.7

## Data Availability

The data presented in the study are openly available on PhysioNet, accessed on 12 June 2022, at https://doi.org/10.13026/7vcr-e114.

## Abbreviations

The following abbreviations are used in this manuscript:

Lasso	least absolute shrinkage and selection operator
ICU(s)	intensive care unit(s)
SVM	support vector machine
MIMIC	the medical information mart for intensive care
BIDMC	the Beth Israel Deaconess Medical Center
QL	lower quartile
QU	upper quartile
MCAR	missing completely at random
MAR	missing at random
Logit	logistic regression
ROC	receiver operating characteristic
AUC	area under the curve
GVIF	generalized variance inflation factor
AGVIF	adjusted generalized variance inflation factor
ACC	accuracy
TP	number of true positive samples
FP	number of false positive samples
TN	number of true negative samples
FN	number of false negative samples
